# Spine plasticity to restore the cortical networks of movement: a therapeutic approach to spinal cord injury

**DOI:** 10.1093/braincomms/fcaf076

**Published:** 2025-03-13

**Authors:** Caroline Machado, Edmund Hollis

**Affiliations:** Burke Neurological Institute, White Plains, NY 10605, USA; Burke Neurological Institute, White Plains, NY 10605, USA; Feil Family Brain and Mind Research Institute, Weill Cornell Medicine, White Plains, NY 10065, USA

## Abstract

This scientific commentary refers to ‘Edonerpic maleate enhances functional recovery from spinal cord injury with cortical reorganization in non-human primates’, by Uramaru *et al*. (https://doi.org/10.1093/braincomms/fcaf036).

**This scientific commentary refers to ‘Edonerpic maleate enhances functional recovery from spinal cord injury with cortical reorganization in non-human primates’, by Uramaru *et al*. (https://doi.org/10.1093/braincomms/fcaf036).**

One of the principal challenges in translational neuroscience has been to establish therapeutic strategies that support the recovery of movement after central nervous system injuries. Stroke, traumatic brain injury and spinal cord injury severely impact quality of life in survivors and are principal drivers of disability adjusted life years globally in those affected by neurological disorders.^1^ Unlike stroke and traumatic brain injury that can cause direct damage to the cortex, following spinal cord injury cortical substrates remain intact. While supraspinal substrates remain undamaged, alterations in input and output drive change in the cortical circuits that control movement.

The sensory and motor cortex regions that control movement are organized in a rough topography that is shaped through the alteration of both intracortical connectivity and corticofugal projections. These cortical maps are refined through motor skill training, with representations of trained muscles expanding into neighbouring cortical substrates, a process conserved across rodent and non-human primate models.^2,3^ Spinal cord injury severely disrupts these cortical maps, driving long-lasting expansions of areas above the level of injury at the expense of those below.^4^

Changes in cortical maps occur within hours after spinal cord injury in both humans and animal models.^5^ Over the days that follow, this absence of output leads to a loss of dendritic spines in corticofugal pyramidal neurons. Within layer 5b, both injured and uninjured neurons show reduced dendritic spine density in the first week following injury.^6,7^ Over the next weeks to months, spine remodelling results in reduced stable spines and increased numbers of immature, filopodia-like spines.^7,8^ These dramatic changes in spine density and maturity are likely to govern the reduced cortical map areas seen following spinal cord injury as local network connectivity weakens.

In this context, edonerpic maleate represents a potential therapeutic intervention for enhancing cortically mediated movement recovery. The research team behind the current study^9^ previously demonstrated that edonerpic maleate acts by binding collapsing-response-mediator-protein 2 (CRMP2) and facilitating the delivery of glutamatergic AMPA receptors to synapses, thereby strengthening synapses and enhancing circuit plasticity.^10^ Plasticity within cortical maps appears to be a key component of movement recovery as it accompanies spontaneous or rehabilitation-driven gains in recovery after spinal cord injury.^5^ While the plasticity of cortical networks may indicate novel connectivity within the spinal cord from injured or sprouting descending circuits,^11^ it must include some level of synaptic remodelling within the cortex itself if novel spinal circuits are to be integrated into functional cortical networks.

As map remodelling coincides with functional recovery or adaptation from spinal cord injury, enhancing this circuit reorganization is a particularly attractive target for supporting the development of therapeutic strategies to promote recovery. Previously the team found that edonerpic maleate was able to open a window of plasticity in rodent and non-human primate models of cortical injury, supporting the return of precision reach, a key function of the corticospinal tract.^10^ To explore the effect of edonerpic maleate after spinal cord injury affecting the corticospinal tract, Uramaru *et al*.,^9^ in their recent article in *Brain Communications* evaluated the recovery of trained non-human primates on forelimb tasks that required precise grip and motor control. Edonerpic maleate facilitated the recovery of precision grip, while two-thirds of control animals attempted an alternative underhand strategy to compensate for the loss of digit control. Furthermore, cortical mapping after rehabilitation demonstrated an increase in cortical wrist representations in edonerpic maleate-treated animals, mirroring improvements in task success and wrist dorsiflexion (Fig. 1). These effects may be driven by cortical plasticity mechanisms, by local axonal plasticity of residual axons, or by some combination of the two. Corticospinal axons in non-human primates have been shown to exhibit high levels of sprouting after spinal cord injury, though not direct circuit regeneration.^12^ Effectively strengthening and appropriately shaping the residual circuits that drive movement represents a critical strategy for supporting recovery after spinal cord injury.

**Figure 1:**
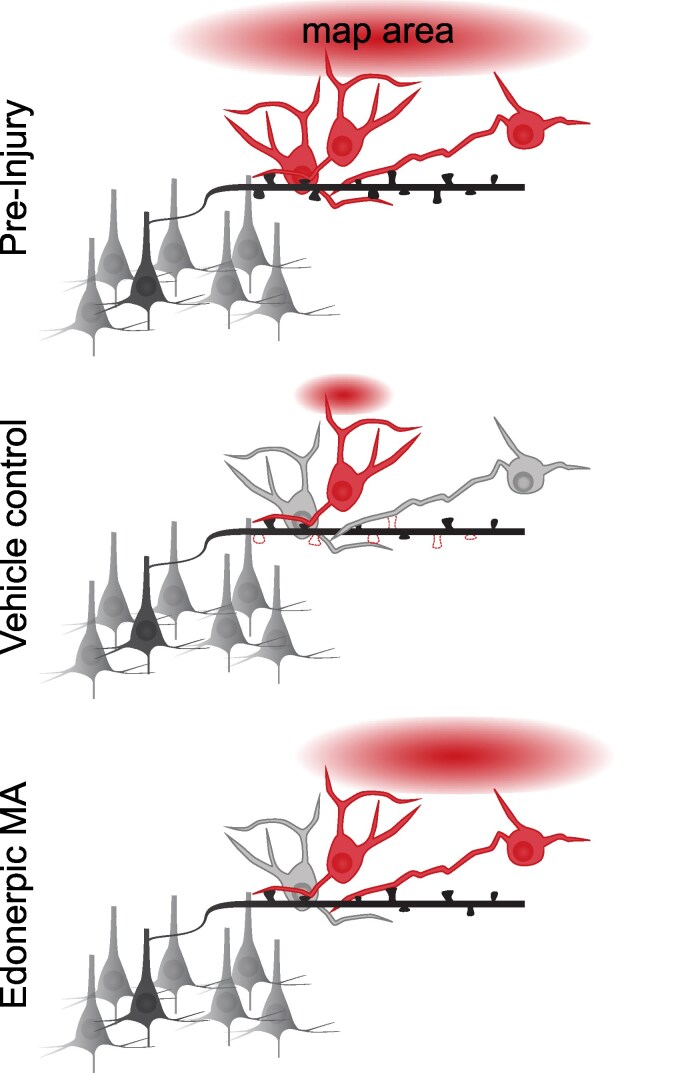
**Dendritic spine stabilization by edonerpic maleate as a mechanism to restore cortical maps after spinal cord injury**. Spinal cord injury leads to a loss of dendritic spines (dashed red lines) within layer 5 cortex and reduced map representations (red oval) from affected limbs. The ability for edonerpic maleate to increase AMPAR localization to dendritic spines may strengthen or stabilize spines reduced by injury. This spine plasticity could restore affected cortical motor networks and provide a substrate for enhancing recovery of movement.

Most spinal cord injuries are anatomically incomplete, even in the complete absence of voluntary movement control. This is apparent as strong electromyography responses can be evoked in clinically affected muscles using transcranial magnetic brain stimulation.^13^ Engaging these residual descending pathways using epidural spinal stimulation allows for chronically injured individuals to initiate voluntary movements.^14^ Enhancing circuit plasticity with modulators like edonerpic maleate may prove to be an effective intervention for restoring function lost to neurological injury and the successful application in multiple models of central nervous system injury make it an appealing translational candidate. While translation of therapeutics from animal models to the clinic is particularly challenging in neurology, edonerpic maleate has already passed an important bar in being proven safe and tolerable in a phase 2 randomized, double-blind clinical trial for Alzheimer’s Disease.^15^ Combinatorial treatments aimed at strengthening spared pathways and re-training the cortical networks required for voluntary movement may lead to long-lasting improvements in recovery and quality of life for those living with SCI.

## Data Availability

No data was generated for this commentary.
